# Impact of Transcranial Direct Current Stimulation (tDCS) on Neuronal Functions

**DOI:** 10.3389/fnins.2016.00550

**Published:** 2016-11-30

**Authors:** Suman Das, Peter Holland, Maarten A. Frens, Opher Donchin

**Affiliations:** ^1^Department of Biomedical Engineering and Zlotowski Center for Neuroscience, Ben Gurion University of the NegevBe'er Sheva, Israel; ^2^Department of Neuroscience, Erasmus MCRotterdam, Netherlands; ^3^Department of Integrative Neurophysiology, Center for Neurogenomics and Cognitive Research, Vrije Universiteit AmsterdamAmsterdam, Netherlands; ^4^Faculty of Social and Behavioral Sciences, Erasmus University College, Erasmus UniversityRotterdam, Netherlands

**Keywords:** tDCS, plasticity, neurotransmitters, neuromodulators, oscillation

## Abstract

Transcranial direct current stimulation (tDCS), a non-invasive brain stimulation technique, modulates neuronal excitability by the application of a small electrical current. The low cost and ease of the technique has driven interest in potential clinical applications. However, outcomes are highly sensitive to stimulation parameters, leading to difficulty maximizing the technique's effectiveness. Although reversing the polarity of stimulation often causes opposite effects, this is not always the case. Effective clinical application will require an understanding of how tDCS works; how it modulates a neuron; how it affects the local network; and how it alters inter-network signaling. We have summarized what is known regarding the mechanisms of tDCS from sub-cellular processing to circuit level communication with a particular focus on what can be learned from the polarity specificity of the effects.

## Introduction

Transcranial direct current stimulation (tDCS), a safe (Bikson et al., 2016) non-invasive brain stimulation technique, has beneficial effects in a range of neurological disorders (Fregni et al., 2015). Positive results have been reported in stroke (Gomez Palacio Schjetnan et al., 2013; Peters et al., 2016), Alzheimer's disease (Boggio et al., 2009), movement disorders (Benninger et al., 2010), depression (Blumberger et al., 2015), schizophrenia (Brunelin et al., 2012), and addiction (Dunlop et al., 2016). Growing use of tDCS creates pressure to understand the underlying mechanisms and, thus, to enable optimal application (Dubljević et al., 2014). In parallel, reproducibility of tDCS effects has been weak in some behaviors (Gladwin et al., 2012; Lally et al., 2013; Wiethoff et al., 2014). Some have suggested that too few tDCS studies test effects at the individual level, reproducible within an individual, in a double-blind design (Horvath et al., 2014). In a meta-analysis, the same group claimed than combining data across studies eliminates the statistical significance of the effect of tDCS on almost all measures of brain activity (Horvath et al., 2015). A clear understanding of the mechanisms through which tDCS may have its effects is conspicuously necessary.

**Figure 1:**
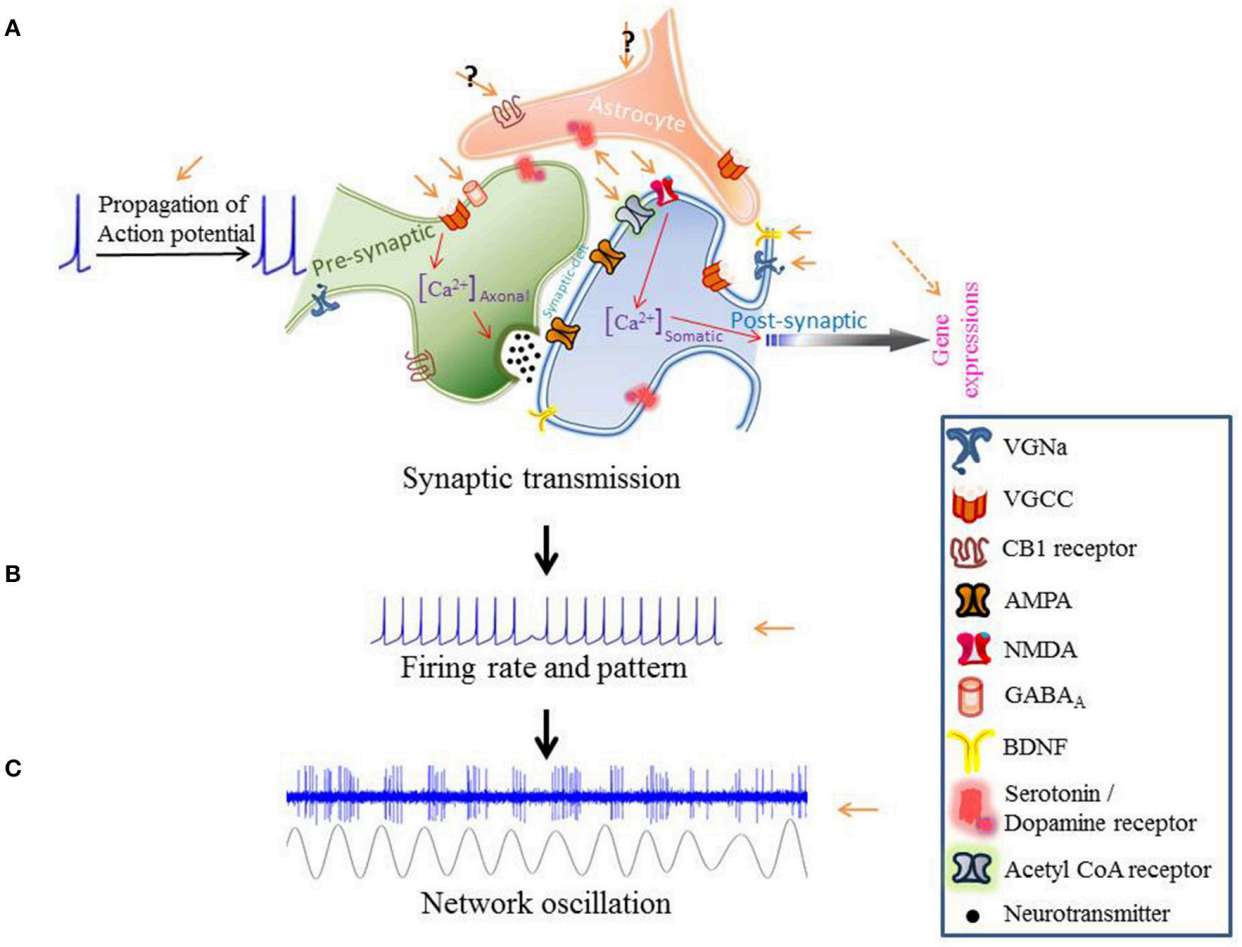
**The modulatory effects of tDCS from subcellular processing to the circuit level communication**.

Transcranial direct current stimulation (tDCS) effects change in the activity of individual neurons: changes in neuronal firing rate and pattern or modulations in synaptic release probability, uptake and sensitivity (Thorpe et al., 2001; Takemura and Kawano, 2002). We focus on the mechanisms of tDCS from sub-cellular processing to circuit level communication (Figure 1). Where appropriate, we will look at how these mechanisms might influence behavior.

## Effects of electrode polarity and placement on neuronal response

tDCS modulates neural activity by applying a weak constant electrical current (amplitude <2 mA) through scalp electrodes (Stagg and Nitsche, 2011). Anodal tDCS (atDCS) refers to the application of positive current whereas cathodal tDCS (ctDCS) applies negative current to the target. The response of an individual neuron to current depends on distance from the current source, orientation with respect to the electrical field and morphology of the neuron.

Distance from the stimulating electrode can alter the polarity specific effects in the target region. For instance, in cerebral cortex of anesthetized rodents, atDCS increased the spontaneous firing and the number of active units close to the electrode (depth <500 μm) whereas cathodal tDCS (ctDCS) reduced the spontaneous firing (Stagg and Nitsche, 2011). The effects persisted for more than an hour after stimulation. In contrast, neurons in deep cortical layers were often deactivated by atDCS and activated by ctDCS (Purpura and Mcmurtry, 1965). This difference may be because intensity varies with distance from the electrode. However, studies in isolated turtle cerebellum (Chan et al., 1988), rodent hippocampal slice (Bikson et al., 2004) and ferret visual cortex slice (Fröhlich and McCormick, 2010) demonstrated no polarity-reversal with varying intensity. Rather, the field strength altered the membrane voltage linearly up until the point that stimulation led to the generation of action potentials. We propose that polarity-reversal after a specific depth may be due to either differences in the lateral connections of neurons or cortical current source density (CSD) (Rappelsberger et al., 1982) distribution, rather than a decrease in current intensity. The CSD depth profile in human neocortex showed a maximal source (outward current) in layer I and sink (inward current) in layers II/III during oscillatory activity (Csercsa et al., 2010). Possibly, atDCS strengthens this dipole formation and thereby neurons in the deeper layer show decrease in activity. However, we must be careful in connecting diverse animal and human studies before reaching a conclusion.

The orientation of neurons may be a significant factor in determining the effects of tDCS. Pyramidal neurons (dendro-axonic orientation) parallel to the current field were activated by atDCS and inhibited by ctDCS (Bindman et al., 1964). Similarly, in the cerebellar cortex maximal modulation was in Purkinje cells (PC) and stellate inter-neurons with a dendro-axonic orientation parallel to the current vector (Chan and Nicholson, 1986); apical dendrites of the PC were depolarized while the rest of the dendrites and soma were hyperpolarized during atDCS (Chan et al., 1988). Conversely, ctDCS depolarized the soma and hyperpolarized apical dendrites. Furthermore, Kabakov et al. (2012) revealed that the field-excitatory post-synaptic potential (fEPSP) on hippocampal slices was maximally suppressed when the action potential traveled toward the cathode and was either facilitated or remained unchanged when propagated toward the anode. Overall, axonal orientation seems to determine whether stimulation is excitatory or inhibitory whereas dendritic orientation governs the magnitude of the stimulation effect.

The morphology (size and structure) of neurons affects the magnitude of the effect. Polarity specific modulation was higher in pyramidal neurons than non-pyramidal neurons in feline encephale isole preparation (Purpura and Mcmurtry, 1965). Moreover, the soma of the largest cortical neurons, layer-V pyramidal neurons, was depolarized the most by atDCS in a rodent slice preparation (Radman et al., 2009). These results imply that soma volume affects tDCS effect magnitude. Apparently, dendritic structure also affects response magnitude. For instance, maximal atDCS polarization is at the tips of basal and apical dendrites of CA1 neurons (Bikson et al., 2004) which may reflect the effect of passive cable properties on the effects of tDCS. Human studies also confirm that atDCS effects on cortical excitability depend on position, size and orientation of the electrodes (Opitz et al., 2015; Ho et al., 2016). However, standardization of the stimulation amplitude requires serious attention (Rampersad et al., 2014).

In summary, these data suggest that polarity specific effects of tDCS depend on distance from the stimulation electrode, current gradient, pre-synaptic axonal orientation, post-synaptic dendritic orientation and neuronal morphology.

## Effects on intracellular plasticity mechanisms

One of the most exciting effects of tDCS has been the ability to modulate the rate of learning in motor adaptation tasks (Jayaram et al., 2012; Herzfeld et al., 2014). tDCS may potentiate learning by affecting the intracellular Ca^2+^ concentration (Figure 1A).

atDCS of cerebral cortex and hippocampus increased intracellular Ca^2+^ concentration (Islam et al., 1995a; Bikson et al., 2004). A rise in intracellular Ca^2+^ concentration drives short and long-term plasticity (LTP) (Greer and Greenberg, 2008). Interestingly, cerebellar atDCS led to Ca^2+^ spikes. Interestingly, Chan and Nicholson (1986), documented cerebellar neurons activated by both atDCS and ctDCS. These neurons generated Na^+^ spikes during atDCS and Ca^2+^ spikes during ctDCS (Chan et al., 1988). This region specific Ca^2+^ spiking is characteristic of the complexity of the effects of tDCS stimulation and the difficulties involved in interpreting results.

The exact mechanism underlying the increase in intracellular Ca^2+^ remains under investigation and it is possible that tDCS may target voltage dependent Ca^2+^-channels. In the presence of N-methyl-D-aspartate (NMDA) blockers, Ca^2+^ dependent expression of early gene (c-fos) on the atDCS side was absent, except around the polarized point itself (Islam et al., 1995b). Hippocampal slice studies also showed residual changes in Ca^2+^ levels, even in the presence of NMDA blockade (Bikson et al., 2004). This has fed speculation of an alternative mechanism that is dependent on voltage-sensitive Ca^2+^ channels (VGCC). Recently, Christie et al. (2011) showed that sub-threshold somatic depolarization was sufficient to activate axonal VGCCs that elicited Ca^2+^ influx. These animal studies indicate that atDCS opens Ca^2+^ channels by increasing transmembrane potential. Furthermore, higher intensity and longer duration atDCS has greater effects on Ca^2+^ accumulation (Islam et al., 1995a). Additionally, an *in vivo* mouse experiment showed that tDCS elevates astrocytic Ca^2+^ surges that promotes cortical metaplasticity (Monai et al., 2016). Comparing Ca^2+^ regulation at the cellular level in humans and animals is complicated because the distribution of channel subtypes is species and region specific (McKay et al., 2006). However, in humans, NMDA channel antagonists also abolished atDCS effects (Liebetanz et al., 2002; Nitsche et al., 2003) whereas agonists enhanced those (Nitsche et al., 2004b). Similarly, Ca^2+^ channel blockers selectively eliminated atDCS enhancement of cortical excitability (Nitsche et al., 2003) suggesting that VGCC may facilitate tDCS driven Ca^2+^ accumulation in humans as in animals.

## Effects on neurotransmission

The excitability of a neuronal network can be modified by modulating neurotransmitter release-probability or receptor-affinity. tDCS could change the rate of neurotransmitter release either through effects on action potential propagation or vesicle release probability (Figure 1A). Receptor-affinity modulation could be achieved by engaging neuromodulators.

There is strong evidence that tDCS affects neurotransmitter concentration. Primary motor-cortex atDCS reduced local gamma-aminobutyric acid (GABA) concentration (Stagg et al., 2009; Kim et al., 2014) whereas ctDCS reduced both glutamate (Glu) and GABA concentrations in human cortex (Stagg et al., 2009). In contrast to Stagg et al. (2009), in which stimulation was performed at rest, Kim et al. (2014) observed no ctDCS effect on GABA when subjects were performing a motor adaptation task. Therefore, tDCS effects on neurotransmitter concentration may be task specific. Supporting this, an activity dependent GABA_A_ agonist blocker eliminated motor evoked potential (MEP) facilitation by atDCS (Nitsche et al., 2004c). However, in a more recent study atDCS over primary-motor cortex had no effects on GABA concentration and receptor activity in either healthy or with mild Traumatic Brain Injury patients (Wilke et al., 2016).

One hypothesis to consider is that atDCS might increase both Glu and GABA levels. The mechanisms for this could be sub-threshold depolarization or network oscillation. For instance, sub-threshold depolarization of the cerebellar molecular layer inter-neurons (MLIs) enhanced GABA release (Christie et al., 2011). Subthreshold oscillations in the dendrites of mitral cells in the accessory olfactory bulb are coupled to dendritic Glu release (Castro and Urban, 2009). Thus, if both mechanisms are activated, atDCS may actually increases both Glu and GABA release. This would provide a mechanism for atDCS dependent neuronal synchronization (Figure 1B). However, this hypothesis combines evidence taken from different experiments performed in different brain regions with different methodologies. A focused investigation would be necessary to give this speculation concrete support.

## Neuromodulators and tDCS

tDCS can affect synaptic neuromodulator concentration. Conversely, the concentration of a neuromodulator, by affecting synaptic dynamics, can change the effect that tDCS has on that synapse (Figure 1A).

tDCS and serotonin enhance each other's function. For instance, atDCS reduced the symptoms of major depressive disorders (Murphy et al., 2009), in which the serotonergic system is compromised (Morrissette and Stahl, 2014). Moreover, the effects of tDCS on the serotonergic system seem to be mediated by specific variants of the serotonin transporter (5-HTTLPR) (Brunoni et al., 2013). We, therefore, speculate that genetic polymorphism contributes to the individual sensitivity toward tDCS. Plausibly, this is the reason for inter-subject variability in tDCS dependent MEP modulation (Wiethoff et al., 2014). Incremental increases in extracellular serotonin levels, using selective serotonin reuptake inhibitor (SSRI), boost anodal facilitation of MEP and caused ctDCS to have an excitatory effect (Nitsche et al., 2009). Moreover, atDCS of the temporal cortex improved memory formation when serotonergic neurotransmission was enhanced simultaneously (Prehn et al., 2016). Thus, tDCS magnifies the activity of serotonergic system. No existing models explain how serotonin might reverse the cathodal and enhance the anodal effects of tDCS. Nevertheless, the evidence does support a bidirectional relationship: atDCS promotes the function of the serotonergic system and serotonin facilitates atDCS effects.

It is possible that tDCS modulates skill learning by altering brain-derived neurotrophic factor (BDNF) dependent cortical plasticity. This notion was validated by an *in vitro* M1 study in which the atDCS promoted BDNF-dependent LTP (Fritsch et al., 2010). It is plausible that tDCS: (i) enhances secretion of BDNF which influences the spike-time dependent plasticity (Tanaka et al., 2008) and, (ii) modulates the BDNF mediated late-phase of plasticity (Pang et al., 2004). The BDNF Val66Met polymorphism in humans may also be a factor in determining the individual sensitivity to tDCS (Puri et al., 2015).

Other neuromodulators appear to have more complex effects. For instance, a dopamine (DA) agonist turned the atDCS facilitation of motor cortex into inhibition and prolonged the usual ctDCS inhibition (Kuo et al., 2007) in humans. Thus, DA effects on tDCS are precisely opposite to those of serotonin. Interestingly, atDCS of the frontal cortex improved short-term memory by elevating dopaminergic neurotransmission in the rodent hippocampus and striatum (Leffa et al., 2016). Nicotine (Thirugnanasambandam et al., 2011) and cholinesterase-blockers (Kuo et al., 2008) both had the effect of abolishing both anodal and cathodal effects on primary motor cortex in humans. Amphetamine enhanced and prolonged the anodal effects (Nitsche et al., 2004a), but has not been tested in ctDCS. Significant reduction in anodal after-effect could be observed by administration of a β-receptor antagonist. All in all, clinical application of tDCS will require awareness of the potential interactions and also the influences of specific genetic backgrounds.

## Modulation of brain oscillations

Empirically, alterations in neural oscillations have been found in all major psychiatric diseases (Buzsáki and Watson, 2012). The hope is that tDCS could provide clinical relief by strengthening or weakening oscillatory activities within brain regions (Figure 1C).

tDCS induces transient and reversible effects on high-frequency beta and gamma oscillations. ctDCS significantly decreased visually evoked oscillations at these frequencies whereas atDCS led to a slight increase (Antal et al., 2004). Simultaneous oppositely polarized stimulation of both agonist and antagonist cortical hand movement regions (with the agonist stimulated anodally) led to increase in gamma activity in functionally connected regions during movement (Polanía et al., 2011). Both of these studies showed an enhancement in high frequency oscillations following atDCS.

Similarly, ctDCS suppressed (and atDCS enhanced) gamma oscillations in rodent hippocampus (Reato et al., 2010) and ferret visual cortex (Fröhlich and McCormick, 2010). atDCS increased oscillatory frequency by shortening the duration of the Down-state but not the Up-state of multi-unit activity. Longer atDCS could also induce lasting effects in gamma oscillations (Reato et al., 2015). In summary, (i) tDCS can modulate synchronization and topological functional organization of the brain by altering specific frequency bands and (ii) in active neuronal networks, atDCS induces long-lasting facilitatory effects on high frequency oscillations. tDCS induced gamma modulation may be a suitable method to promote higher order cognitive processes in certain neurological diseases.

## Spatial extent

Application of tDCS over a specific brain region induces neuronal modulation not only in that region but also to its downstream structures (Li et al., 2015). atDCS of the rodent frontal cortex enhanced neuronal activity there and also in the nucleus-accumbens (Takano et al., 2011). atDCS of rodent cortex led to increased intracellular Ca^2+^ accumulation (Islam et al., 1995a) and early gene expressions (Moriwaki et al., 1995; Islam et al., 1995b) in the ipsilateral connected cortical and subcortical regions. Strikingly, ipsilateral atDCS on the ischemic cortex in a rodent stroke model led to dendro-axonal growth in both hemispheres (Yoon et al., 2012). The combined intervention of anodal and ctDCS on contralateral sides changed the intra-hemispheric and the inter-hemispheric topological functional organization and the intra-cortical synchronization in human (Polanía et al., 2011). These studies all argue that tDCS effects are not completely focal.

Despite evidence for effects on additional structures, behavioral studies usually indicate a focal effect. Psychomotor performance improved with atDCS of the facilitatory loop (the circuit whose activity promotes a behavior) and/-or with ctDCS of the competitive loop (the circuit whose activity hinders a behavior) (Vines et al., 2008). One measure of focal specificity is the minimum distance between stimulating electrodes that produce the same behavioral effect. atDCS of the cerebellum but not M1 facilitated visuomotor (Galea et al., 2011) and force field (Herzfeld et al., 2014) adaptation. Thus, tDCS can distinguish anatomically well separated targets. Left M1 atDCS induced relatively greater improvement in right handed motor skill than right M1 stimulation (Schambra et al., 2011). At a much finer scale, atDCS of the left supplementary motor area (SMA) and M1 both led to improvement in a visuomotor skill task but left pre-SMA stimulation did not (Vollmann et al., 2013). High-definition tDCS promises to allow stimulation of subparts of cortical sub-regions (Villamar et al., 2013). Hence, despite effects in connected regions tDCS has potential as a focal non-invasive brain stimulation technique in neuro-rehabilitation.

## Temporal extent

Long lasting offline (post-stimulation) effects are crucial for effective intervention. Thus, the effectiveness of tDCS is questioned not only in terms of its specificity but also in terms of the extent of offline effects.

Effects persisting for an hour after cessation of stimulation have been reported in terms of firing rate (Bindman et al., 1964), fEPSP (Fritsch et al., 2010) and gamma-oscillations (Reato et al., 2015) in rodent cortex. Similarly, a meta-analysis claimed that tDCS on human has an offline neurophysiological effect only on MEP amplitude modulation (Horvath et al., 2014). Similar findings for neuromodulators are limited as the interactions between neuromodulators and tDCS were measured through drug administration that had acute receptor saturation and washout effects (Kuo et al., 2008; Nitsche et al., 2009; Thirugnanasambandam et al., 2011). One key complicating issue, highlighted in this review, is the multiplicity of mechanisms through which tDCS may work across brain regions. Focusing in on how tDCS might have an offline effect, there a few cellular mechanisms that might mediate it-intracellular Ca^2+^ concentration (Islam et al., 1995a; Bikson et al., 2004) and early gene expressions (Moriwaki et al., 1995). Unfortunately, the available studies do not provide temporal data on offline effects.

A few behavioral experiments have explored offline effects of tDCS. atDCS paired to learning facilitated locomotor (Jayaram et al., 2012), force field (Herzfeld et al., 2014) adaptation and eye-blink conditioning (Zuchowski et al., 2014) tasks. Surprisingly, post-stimulation deadaptation curves (Jayaram et al., 2012; Herzfeld et al., 2014) or extinction rate (Zuchowski et al., 2014) showed no polarity specific differences.

In summary, we can say that it is just too early to declare anything clear about online and offline effects of tDCS on either a cellular or a behavioral level. Moreover, offline effects of tDCS are not very consistent across the various paradigms tested so far.

## Conclusion

Future experiments studying polarity specific effects of tDCS on the brain need to accomplish a detailed monitoring and manipulation of cellular and sub-cellular processes in animals whereas performing tasks that optimally engage (and differentiate) brain states and regional associations. Such an experiment has yet to be performed, but the recent achievements in this direction reviewed here, give cause for hope that the next couple of years will see significant progress in this endeavor.

## Author contributions

SD, PH, MF, and OD drafted the manuscript. All authors thoroughly went through and approved the current version of this paper.

## Funding

SD, MF, and OD were supported by Marie Curie ITN initiative C7 Research Grant Award. PH is funded by the Marie Curie ITN initiative C7 and Kreitman Post-doctoral fellowship.

### Conflict of interest statement

The authors declare that the research was conducted in the absence of any commercial or financial relationships that could be construed as a potential conflict of interest.
